# Editorial: Low-temperature stress in plants: molecular responses, tolerance mechanisms, plant biodesign and breeding applications

**DOI:** 10.3389/fpls.2024.1411636

**Published:** 2024-05-07

**Authors:** Ping Li, Tangchun Zheng, Douglas S. Domingues, Yang Liu, Sagheer Ahmad

**Affiliations:** ^1^ College of Forestry and College of Landscape and Tourism, Agricultural University of Hebei, Baoding, China; ^2^ School of Landscape Architecture, Beijing Forestry University, Beijing, China; ^3^ Luiz de Queiroz College of Agriculture, University of São Paulo, Piracicaba, Brazil; ^4^ Biosciences Division, Oak Ridge National Laboratory, Oak Ridge, TN, United States; ^5^ Key Laboratory of National Forestry and Grassland Administration for Orchid Conservation and Utilization at College of Landscape Architecture, Fujian Agriculture and Forestry University, Fuzhou, China

**Keywords:** low-temperature, abiotic stress, molecular responses, plant biodesign, breeding, genome-editing, membrane lipids

Low temperature stress is a major factor that affects the normal growth, development, and geographical distribution of plants, and has always been a hot topic in plant research. Against the backdrop of rapid development in biological breeding, deciphering the molecular mechanisms underlying plant responses to low temperature stress has become particularly urgent. In order to further refine the molecular mechanisms underlying plant responses to low temperature stress, this Research Topic of *Low-temperature stress in plants: molecular responses, tolerance mechanisms, plant biodesign and breeding applications* has collected seven original research articles and two review articles focusing on essential aspects of this field.

The degree of plant tolerance to low temperature stress varies significantly due to factors such as species, growth environment, and growth stage. Therefore, in the process of studying the molecular mechanisms underlying plant responses to low temperature stress, the selection of plant materials, design of low temperature environments, and evaluation of plant response phenotypic traits to low temperature stress are crucial considerations. The research papers collected on this topic involving seven plant species have significant differences in experimental design due to species variations. Regardless of the strategy used to study the molecular mechanisms underlying plant responses to low temperature stress, summarizing the research on this topic and previous results indicate that the main indicators for evaluating plant response to low temperature stress include ion permeability, soluble sugar content, CAT activity, SOD activity, POD activity, and phenotypic traits associated with plant response to low temperature stress. Yang et al. find that low temperature stress significantly affects the yield and quality of rice grains during the grain-filling phase. In Song et al. study, Korean pine (*Pinus koraiensis* Siebold & Zucc) and Simon poplar (*Populus simonii* Carr.) trees were studied, and a detection method suitable for assessing freezing injury in woody plants was developed using electrical impedance spectroscopy (EIS) method. Additionally, the main environmental factors contributing to freezing injury in woody plants were identified.

High-throughput sequencing technology and molecular biology techniques are widely applied in the study of plant responses to low temperature stress. Integrating the research findings of Elakhdar et al., Wang et al. and Li et al. reveals the complex molecular mechanisms underlying plant responses to low temperature stress, which involve the coordinated regulation of multiple pathways. Among them, the widely studied pathways involved in these studies include the Ca^2+^ signalling, abscisic acid (ABA) signalling, mitogen-activated protein kinase (MAPK) cascade, and inducer of CBF expression 1 (ICE)-C-repeat binding factor (CBF) signalling pathways (Zhu, 2016; Kidokoro et al., 2022). Additionally, new plant low-temperature responsive genes have been discovered, such as the *CUC2* gene in *Brassica rapa* L and the *COMT*, *CCR*, *CAD*, *PER* and *F3’H* gene in *Prunus persica* L. Batsch in Tao et al. and Li et al. studies. The discovery of these low-temperature responsive genes in plants also provides genetic resources for molecular breeding of cold tolerance in plants. The role of non-coding RNAs in regulating plant responses to low temperature stress has been studied in *japonica* rice. Wang et al. unveils cold tolerance-related competing endogenous RNA (ceRNA) networks through whole-transcriptome profiling, identifying key components such as circular RNAs, microRNAs, and messenger RNAs. With WRKY transcription factors playing a pivotal role, the research highlights potential ceRNA networks crucial for enhancing cold tolerance. Meanwhile, previous studies have found that DNA methylation, histone acetylation, and chromatin accessibility play important roles in plant responses to low temperature stress (Ding et al., 2020). Therefore, it can be seen that molecular interactions and epigenetics are also important factors involved in plant responses to low temperature stress.

This Research Topic involves two review articles, Wang et al. providing an overview of the entire plant kingdom in response to  low temperature stress, and Chen et al. focusing on the study of chrysanthemums response to low temperature stress. A comprehensive review of plant responses to low temperature stress from the perspective of the entire plant kingdom reflects the broad-spectrum nature of plant responses to low temperature stress. It provides a macro view, including topics such as plant hormone regulation, epigenetic modifications, and multiple molecular regulatory pathways. On the other hand, a specific species-focused review on chrysanthemums response to low temperature stress showcases more specific research methods and experimental results.

The future prospects of research on plant responses to low temperature stress are promising. With advancements in molecular biology and genetic engineering techniques, we can expect further elucidation of the underlying molecular mechanisms involved in cold tolerance and the development of novel strategies for enhancing low temperature stress resistance in plants. The emerging field of omics technologies, such as genomics, transcriptomics, proteomics, and metabolomics, offers powerful tools for comprehensively studying the molecular responses of plants to low temperature stress. By understanding the molecular basis of cold tolerance, researchers can potentially manipulate these genes to enhance the ability of plants to withstand freezing temperatures. Furthermore, the application of advanced breeding techniques, such as marker-assisted selection and genome editing, holds great promise for improving low temperature tolerance in crops. By precisely targeting specific genes or genetic regions associated with cold tolerance, breeders can expedite the development of new varieties with enhanced resilience to low temperatures. Considering the current research on plant responses to low temperature stress, the major challenge lies in the plant genetic transformation system, particularly for non-model plants. Addressing this issue is crucial for studying the molecular mechanisms of plant responses to low temperature stress and accelerating the development of new varieties with enhanced cold tolerance. Therefore, to expedite the progress of molecular breeding for low temperature tolerance, constructing a plant genetic transformation system is the next urgent scientific problem to be addressed, building upon the research foundation of this Research Topic.

## Author contributions

PL: Data curation, Writing – original draft, Writing – review & editing. TZ: Writing – review & editing. DD: Writing – review & editing. YL: Writing – review & editing. SA: Writing – review & editing.
